# Broadband thin sound absorber based on hybrid labyrinthine metastructures with optimally designed parameters

**DOI:** 10.1038/s41598-020-67688-x

**Published:** 2020-07-01

**Authors:** Yong-xin Gao, Yuan-peng Lin, Yi-fan Zhu, Bin Liang, Jing Yang, Jun Yang, Jian-chun Cheng

**Affiliations:** 1 Key Laboratory of Modern Acoustics, MOE, Institute of Acoustics, Department of Physics, Nanjing University, Nanjing, 210093 People’s Republic of China; 2 Key Laboratory of Noise and Vibration Research, Institute of Acoustics, Chinese Academy of Sciences, Beijing, 100190 People’s Republic of China

**Keywords:** Materials for devices, Acoustics

## Abstract

Broadband acoustic absorbers with thin thickness are highly desired in practical situations such as architectural acoustics, yet it is still challenging to achieve high absorption by using structure with limited thickness. Here we report the theoretical optimal design, numerical simulation and experimental demonstration of a planar acoustic absorber capable of producing broadband sound absorption with deep-subwavelength thickness. The mechanism is that, we use a hybrid design of individual unit cell comprising multiple resonators with a coiled configuration for expanding the working bandwidth and downscaling the resulting device, and, on the other hand, the geometries of the constituent resonance elements are optimally designed by using genetic algorithm. Based on an analytical formula we derive for an efficient prediction of the absorption efficiency, the optimization process is accelerated and gives rise to an optimally maximized amount of absorbed energy with limited device thickness. As a result, the proposed absorber features planar profile, broad bandwidth, wide absorbing angle (the absorber works well when the incident angle of sound wave reaches 60°) and thin thickness (< 1/25 wavelength). In addition, the proposed scheme does not rely on extra sound-absorptive materials or the type of constituent solid material, which significantly simplifies the sample fabrication and improves the application potential of resulting device. The measured data agree well with the theoretical predictions, showing high sound absorption in the prescribed frequency range. We envision our design to further improve the performance of acoustic absorbers and find applications in practical situations in need of elimination of broadband acoustic waves within limited spaces.

Absorption of acoustic energy is important for acoustic research and practical applications such as architectural design and noise control^1,2^. The conventional methods for sound absorption rely on porous and fibrous materials^3–5^ and have to be bulky and heavyweight at low-frequency regime. The recent emergence of acoustic metamaterials^6–15^ and metasurfaces^16–20^ offers sound-absorbing capability as well as flexibility beyond what are available in traditional absorbers^21,22^. Cheng and colleagues propose sawtooth-like metastructures decorated with subwavelength grooves for absorbing broadband airborne sound effectively which still have non-planar profile much thicker than the central working wavelength^23^. Sheng’s group has explored the causality constraint that gives the upper limit of the absorption amount achievable in an acoustic system with certain thickness, and also used a hybrid metamaterial comprising multiple coiled unit cells to verify their theoretical proposal^24^. Their work realized broadband thin acoustic absorber, however, it is still of fundamental interest and practical importance to further increase the total amount of energy absorbed over a wide frequency range with a planar acoustic device of a certain thickness^25^.

In this article, we propose the design of a metamaterial-based acoustic absorber providing optimally high and broadband absorption of incident acoustic energy within limited space. To implement our design, we need a basic unit to construct our material, which can prove the generality and effectiveness of the proposed method. The narrow labyrinthine-like channel, which has a simple configuration and has been proven to be a good candidate for providing high sound absorption effect at resonance^24,26^, is chosen as the constituent unit cell of the sound absorber to be optimized. It is well known that resonant sound absorbers have the ability of providing high-efficiency sound absorption at resonance frequencies. We sum up the absorption coefficient of the total absorber as an efficient calculation and, based on this, use the genetic algorithm^27^ to design the arrangement of absorption peaks in an optimal manner, which is closely related to the geometries of all the resonance elements. The genetic algorithm adopts probabilistic optimization, and does not need to determine rules, automatically acquires search space, and can adaptively adjust search direction, which is a convenient and effective method in our design. As a result, the optimized acoustic absorber bears the advantages of planar profile, thin dimension (thinner than 1/25 of the minimum operating frequency’s wavelength in this particular case) and broadband functionality and can work for a wide range of incident directions. Furthermore, the current scheme needs no additional absorptive materials such as fibrous media necessary and offers the flexibility of choosing the constituent medium that can be freely selected from various solids with sufficiently large acoustic impedance contrast against surrounding air, which significantly facilitates the fabrication of practical devices and ensures the stiffness and environmental-friendliness. We use both numerical simulations and experimental measurements to verify the effectiveness of our proposed scheme.

## Results

**Figure 1:**
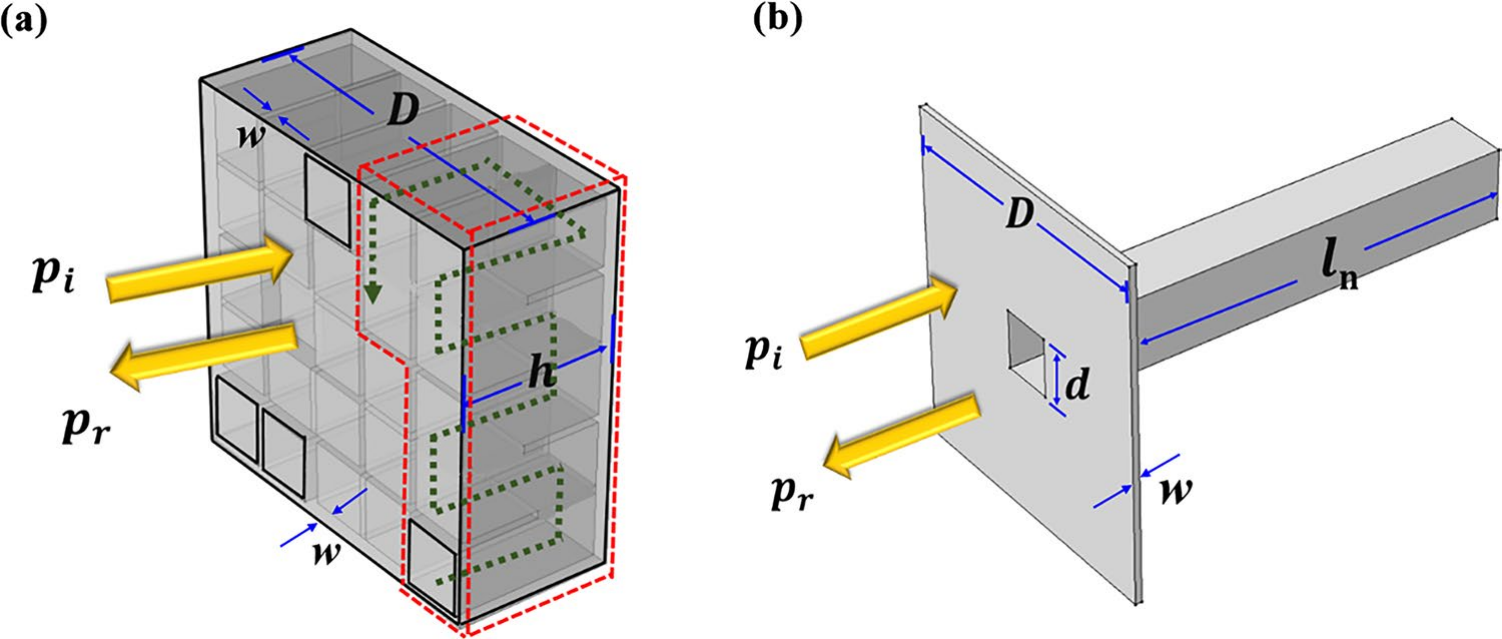
Schematic illustration of an unit cell. (**a**) The schematic configuration of an individual unit cell of the proposed sound absorber which is chosen to consist of 4 resonance elements. The green zigzag line indicates the substantially-elongated propagation distance of incident wave within the *n*th sub-unit cell. (**b**) The typical zoom-in view of a single resonance element.

The schematic of an individual unit cell of the proposed acoustic absorber is shown in Fig. 1a. Each unit cell consists of multiple (chosen as 4 in the current design) resonance elements, with each comprising different numbers of connected square pipes with lateral length of cross-section much smaller than the wavelength. It is well known that as scalar waves, acoustic waves can propagate within such labyrinthine-like pipes in absence of cut-off frequency, which leads to remarkable increase in the total propagation distance^17^ (see the green zigzag line indicating the substantially-elongated propagation distance of incident wave within the sub-unit cell as shown in Fig. 1a). Hence each sub-unit cell can be acoustically regarded as a straight close-ended pipe with identical cross-section yet different lengths, enabling effective interaction with low-frequency sound of wavelength much larger than the physical thickness of the designed absorber. Narrow width of the channel helps minimize the device size so that our coiling material can absorb lower frequency sound with smaller size. Since the width is nearly two orders of magnitude larger than the boundary layer thickness, strong enhancement of energy absorption can be expected at resonance due to dissipation occurring in the opening of cavities.

To simplify the analytical analysis, we consider a model composed of an array of straight channels for which the diameter of the small tube is *d* and the length of the *n*th tube is $l_{n } (n = 1,2,3 \ldots )$, as shown in Fig. 1b. The surface impedance at the opening of the *n*th tube backed with a rigid end can be expressed by $Z_{{\text{n}}} = R_{{\text{n}}} + jX_{{\text{n}}}$. Here $R_{{\text{n}}} = (l_{{\text{n}}} {/}\pi r^{3} )\sqrt {2\eta \omega \rho_{0} }$ and $X_{{\text{n}}} = - \rho_{0} c_{0} {\text{cot(}}kl_{{\text{n}}} {)/}S$ refer to the acoustic resistance and reactance respectively with *r* being the equivalent radius of the small pipe, $\rho_{0} = 1.21\;{\text{kg/m}}^{3}$ is the density of air, $c_{0} = 343\;{\text{m/s}}$ is the acoustic speed in air, $\eta = 1.983 \times 10^{ - 5} \;{\text{Pa}}\;{\text{s}}$ is the dynamic viscosity of air and $S = D^{2}$ is the overall area of an individual unit cell with *D* being its side length. In the current design the ratio between the surface areas of sub-unit cell and the whole unit cell, viz. $d/D$, is chosen as 4(mm)/23(mm). Then one can obtain the sound absorption coefficient for the absorber containing only this resonant element in each unit cell, as $\upalpha = 1 - \left| {r_{{\text{p}}} } \right|^{2}$ where $r_{{\text{p}}} = (Z_{{\text{n}}} - Z_{0} ){/(}Z_{{\text{n}}} + Z_{0} {)}$ is the reflection coefficient of acoustic pressure with $Z_{0} = \rho_{0} c_{0} {/}S$. This sub-unit cell yields a maximum absorption of incident acoustic energy at resonance frequency characterized by $f = c_{0} {/}4l_{n} ,(n = 1,2,3 \ldots )$, where $X_{{\text{n}}} \approx 0$. Similarly, it is readily to derive the equivalent acoustic impedance of the entire unit cell composed of 4 sub-unit cells as $Z_{{\text{t}}} = (\mathop \sum \nolimits_{{{\text{n}} = 1}}^{4} Z_{{\text{n}}}^{ - 1} )^{ - 1}$, and subsequently the total absorption coefficient of the whole absorber as

1 $${\upalpha } = 1 - \left| {\left( {Z_{{\text{t}}} - Z_{0} } \right)/\left( {Z_{{\text{t}}} + Z_{0} } \right)} \right|^{2} .$$

This hybrid design of absorber comprises multiple labyrinthine-shaped resonance elements with different length in one individual unit cell, offering the possibility of simultaneously producing high absorption of incident acoustic wave with a thickness more than one order of magnitude smaller than its wavelength and having a broad working bandwidth. It is noteworthy, however, that the choices of the geometric parameters of these constituent sub-unit cells are closely related to both the physical space occupied by them and their sound absorption capability. Material with narrower channel, thinner thickness does occupy less space, but that will inevitably move the absorption band to higher frequency and lower the absorption of each unit cell. This suggests that there always exists trade-off between the enhancement of absorption of incident energy and downscaling of device footprint in a resonanting sound absorbing structure like the unit cells in our material, and an optimization process is necessary to parallelly select the geometries of all the sub-unit cells for achieving the optimal amount of sound absorption in a broad frequency band by using limited resonant elements with the possibility of mutual influence of spectrum among these sub-unit cells being taken into consideration^26^. Since the value of $d/D$ has already been fixed as 4/23, the device thickness is equivalent to total volume of all the sub-unit cells which is calculated by $h = \left( {\sum l_{n} /25} \right) + 2 \times w$, where $w = 0.5\;{\text{mm}}$ refers to the thickness of the wall of the resonance cavities used in our device. Then we need to design the length of the small pipe, $l_{{\text{n}}}$, for each resonant element, which collectively determine the overall shape of the absorption line. In contrast to the existing designs relying on exponential^24^ or approximately equispaced^26^ distributions of resonance eigen-states in spectrum, we choose to use genetic algorithm, a universal method that has been extensively applied for seeking the optimal solution by simulating natural evolutionary processes, to design the geometry of the sub-unit cells contained in our designed absorber in an optimal manner. The fitness function is chosen as the total amount of absorbed acoustic energy over the frequency range of interest and defined as $A(f) = \mathop \int \limits_{{f_{min} }}^{{f_{max} }} \alpha (f)$, where $f_{max}$ and $f_{min}$ are the upper and lower limits of the target frequency domain. The absorption coefficient at a certain frequency, $\alpha (f)$, can be directly calculated by using our derived analytical expression given by Eq. (1), which serves as a fast method for precisely predicting the absorption functionality of the absorber and is requisite for the acceleration of the optimization process. By using the optimization algorithm, we can find the optimal solutions of the geometries of the four sub-unit cells that gives the maximum of the fitness function. For the particular condition used in the current work, in which the target sound absorption frequency domain is chosen as 1.4–1.9 kHz, the optimal lengths of the four sub-unit cells given by the optimization are 60 mm, 55 mm, 51 mm and 46 mm, which correspond to four eigen frequencies of 1,443 Hz, 1,561 Hz, 1,713 Hz and 1,865 Hz respectively. This physically provides the design of a planar acoustic absorber capable of producing optimal absorption to broadband acoustic wave with subwavelength thickness (< 1/25 wavelength). In our particular case $h = 9.5\;{\text{mm}}$, whereas the actual sample has a thickness $h = 10\;{\text{mm}}$.

**Figure 2:**
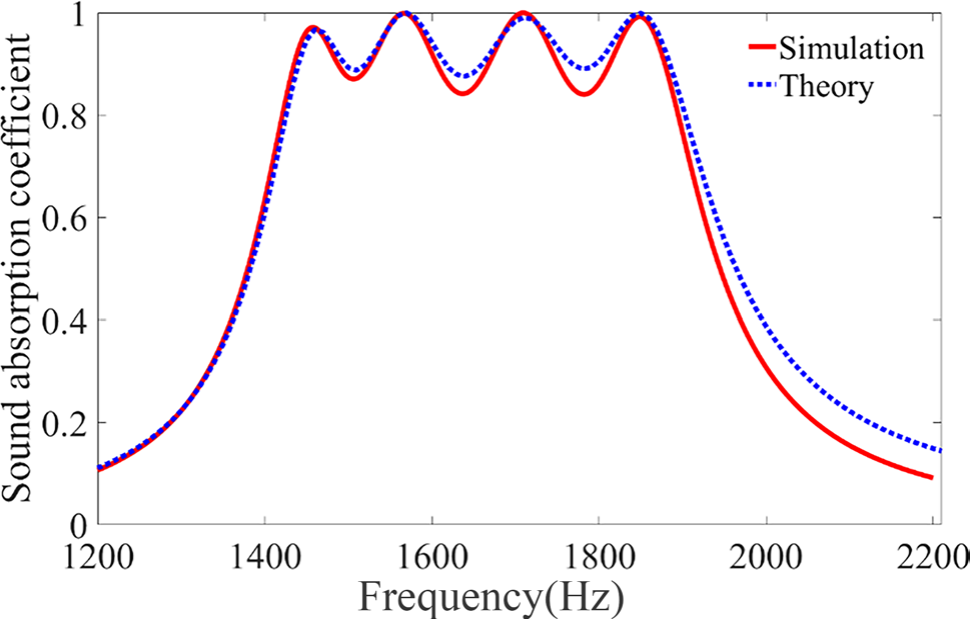
Theoretical and simulation results. Comparison between the frequency dependence of absorption coefficients predicted by analytical formulae and numerical simulation, for an optimized absorber containing four sub-unit cells with their resonant frequencies being 1,443 Hz, 1,561 Hz, 1,713 Hz and 1,865 Hz respectively.

Then we use numerical simulations to verify the accuracy of our design idea. The theoretical and simulated results of the absorption coefficients of our designed absorber under normal incidence of a plane wave are shown in Fig. 2. Good agreement is observed between the theoretical (blue dashed lines) and simulated (red solid line) values, with both showing the occurrence of high absorption coefficient throughout a broad frequency band ranging from approximately 1.4 to 1.9 kHz, which covers the resonance frequencies of all four resonance elements contained in each individual unit cell as expected. Four pronounced resonance peaks are identified at these resonance frequencies, where near-unity absorption of incident acoustic energy can be achieved. It is well known that when the resonance occurs, the acoustic energy of the incident plane wave, which would otherwise distribute uniformly on the sample surface in the absence of resonance, converges inside the corresponding sub-unit cell. As a result of the strong enhancement of the local acoustic field in the resonance element, the sound absorption effect is significantly enhanced despite the weak material dissipation of air. That is to say, the remarkable amplification of the small absorption coefficient by a large local energy density leads to nearly total absorption of incident acoustic energy within the small pipe via the viscous effect of the air. In addition, thanks to our mechanism that use resonance and air viscosity to absorb sound, the resulting absorber needs no additional sound absorption materials and production materials are not limited.

**Figure 3:**
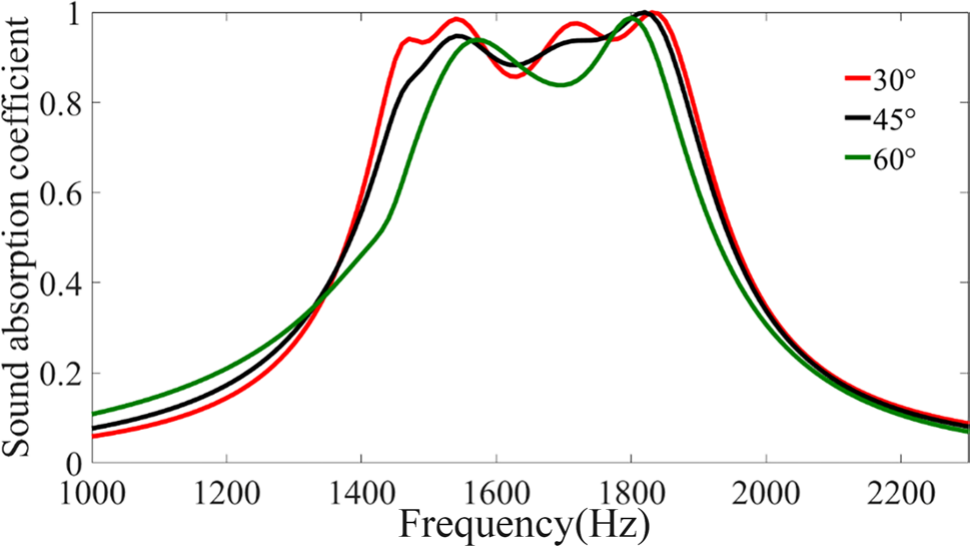
Sound absorption coefficients of incident sound waves at different angles. Comparison of simulated frequency dependence of sound absorption coefficient for three particular directions of incidence: 30°, 45°, and 60°.

Another advantage of our designed absorber is the fact that the absorption performance is insensitive to the change of incident angle which is important for the real-world applications, thanks to the deep-subwavelength nature of the metamaterial unit cells^28^. We have also inspected how the performance of our designed absorber is affected as the angle of incidence varies, and plotted the typical numerical results in Fig. 3 in which three particular angles are considered: 30°, 45° and 60° as marked by red, black and green lines respectively. It is apparent that the designed absorber with optimized parameters can efficiently absorb acoustic wave incident from different angles (albeit the absorption curve deviates slightly from the ideal normal incidence case due to imperfect impedance match at large angles of incidence). Such a wide-angle absorption functionality is ensured by the deep-subwavelength scale of individual resonant elements, which would be of substantial practical significance for the application of our designed absorbers in various scenarios where the acoustic waves usually have random incident directions.

**Figure 4:**
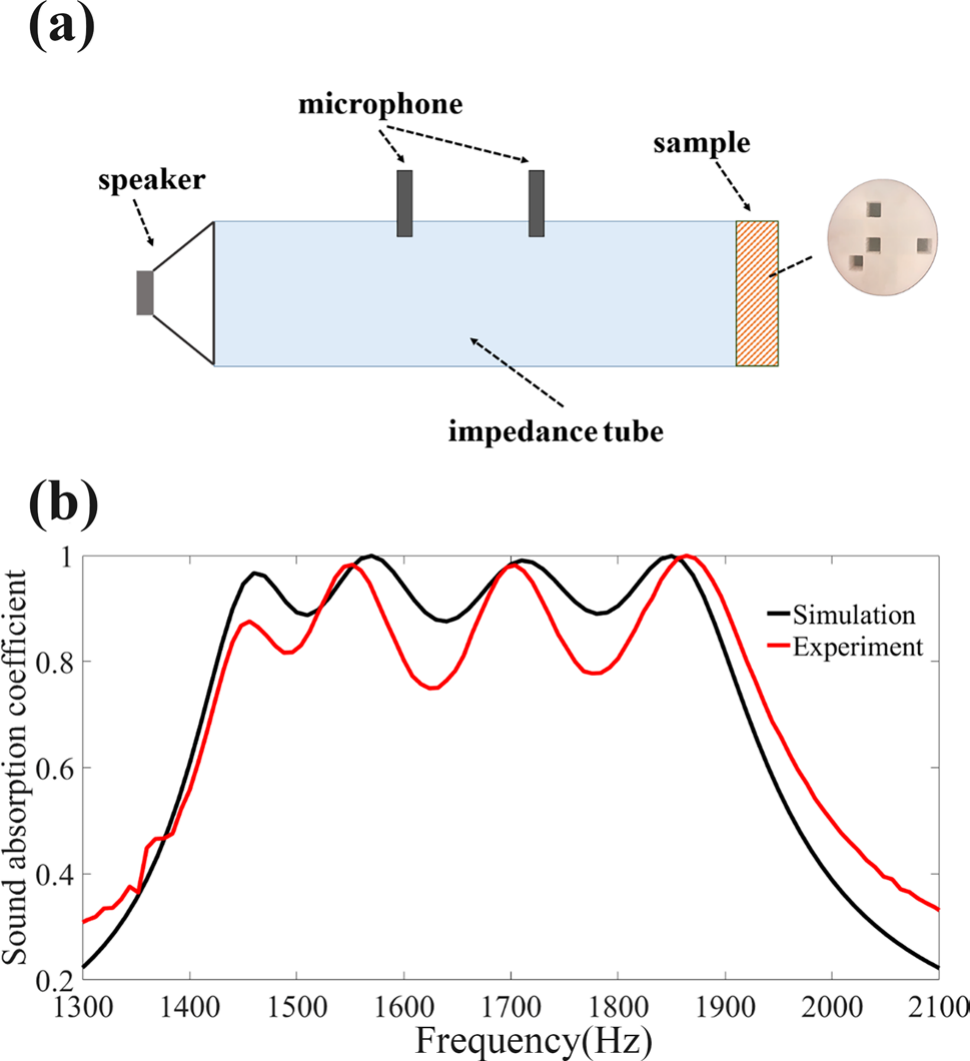
Experimental setup and measured results. (**a**) Schematic illustration of the 2D cross section view of the experimental setup for measuring the sound absorption coefficient of the absorber sample within an impedance tube. (**b**) Simulated and measured absorption coefficients as functions of frequency.

Experiments to verify and demonstrate the sound absorption of our metamaterial were conducted in a commercial impedance tube system, as schematically illustrated in Fig. 4a. For better fitting the geometric shape of the cylindrical waveguide of an inner radius of 15 mm, we fabricated an ultra-thin sample with circular cross section of radius 15 mm via 3-D printing technique (Stratasys Dimension Elite, 0.2 mm in precision). The measured frequency dependence of absorption coefficients is shown in Fig. 4b in which the numerical results of the absorption performance of this specific experimental sample is also provided for comparison. Notice that such a choice of the geometry of absorber, which is not suitable for periodic arrangement for building larger array and thus less applicable in practice, is only for the sake of accuracy and convenience of measurement and does not affect the effectiveness of our proposed mechanism of optimal design of ultra-thin broadband absorbers. The measured data agree well with the theoretical predictions, with both showing the desired broadband absorption functionality of the sample within the prescribed frequency range from 1.4 to 1.9 kHz, despite experimental error which may stem from the imperfect sample fabrication and alignment.

**Figure 5:**
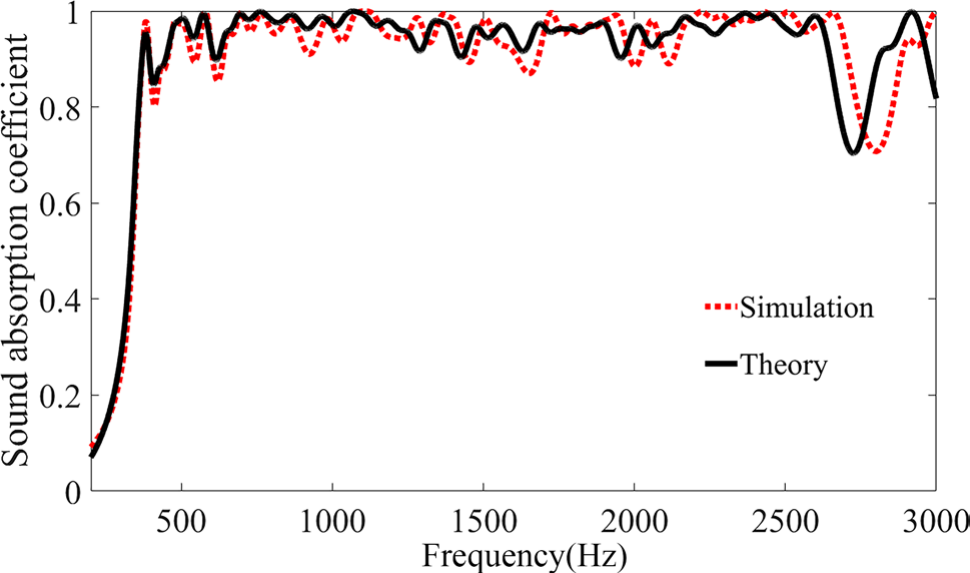
Comparison of frequency dependence of absorption coefficients between the simulation and theory. The black solid line and red dotted line refer to the theoretical and simulated absorption for the structure with structural parameters designed by our proposed method.

When the target frequency domain appears at a lower frequency, our method is still efficient, an example is shown in Fig. 5. In particular, the target sound absorption band corresponding to the result in the Fig. 5 is 350 Hz–2,500 Hz, and $d{/}D = 4\;({\text{mm}}){/}18.5\;({\text{mm}})$, $h = (\sum l_{n} {/}16) + 2 \times w \approx \lambda {/}7$ with $\uplambda$ being the wavelength at 350 Hz.

## Discussion

In summary, we propose a mechanism for designing ultra-thin acoustic absorber with high broadband absorption. We use a hybrid design of unit cells consisting of multiple labyrinthine channels that support strong resonances for remarkably enhancing the viscosity-based loss of incident acoustic energy, and derive the analytical formulae that serves as an efficient prediction of its absorption coefficient. Based on this, we employ genetic algorithm to design an absorber with optimized absorption performance, which has the optimal amount of absorbed energy over the frequency range of interest. Thanks to this mechanism, the resulting absorber is endowed with advantages of ultra-thin thickness smaller than 1/25 wavelength in one example, broadband and wide-angle absorption functionality, requiring no additional absorptive materials and offering the flexibility of choosing the constituent solid material. Given the fact that the current designs of acoustic absorbers usually suffer from bulky size, limited bandwidth and dependence of absorptive porous/foam materials, we envision that our method of designing structure parameters of the acoustic absorber by optimization algorithms to further improve the design of broadband and thin sound absorption structures which are highly desired in practical applications such as architectural acoustics.

## Methods

### Numerical simulations

Throughout the paper, the finite element method based on COMSOL Multiphysics software (Acoustic-Thermoviscous Acoustic Interaction module) is used for the numerical simulations. The linearized Navier–Stocks equation is solved and the boundary is set hard. In the numerical simulation, the number of boundary layers is 8, the boundary layer stretching factor is 1.2, and the thickness of first layer is ${\text{d}} = 0.22\;({\text{mm}})\sqrt {100\;({\text{Hz}}){\text{/f}}}$.

### Acoustic measurements

The measurements of sound absorption coefficient were conducted in a commercial impedance tube system (Brüel&Kjær Impedance Measurement Tube Type 4206), as schematically illustrated in Fig. 4a. The absorption coefficient of the sample is obtained by two-microphone method^29^.
